# Thrombotic thrombocytopenic purpura presenting as stroke mimics with normal diffusion-weighted MRI

**DOI:** 10.1186/s12883-023-03489-9

**Published:** 2023-12-11

**Authors:** Ziyi Zhang, Miao He

**Affiliations:** Department of Neurology, The Second Xiangya Hospital, Central South University, Changsha, 410011 Hunan China

**Keywords:** Stroke mimics, Thrombotic thrombocytopenic purpura, Brain MRI

## Abstract

**Background:**

Thrombotic thrombocytopenic purpura (TTP) is a rare and fatal thrombotic microangiopathy-based hematologic disease. Stroke has been reported as atypical neurological manifestations of TTP in some cases.

**Case presentation:**

A 65-year-old male presented with typical acute ischemic stroke symptoms including sudden-onset dysarthria, right-sided facial paralysis and hemiplegia. However, his CT and MRI scans were negative without showing any new ischemic lesions. He was diagnosed with TTP with severe thrombocytopenia, mild anemia, increased LDH, and low ADAMTS-13 activity. The symptoms and positive signs were rapidly resolved after administrating the plasma exchange therapy.

**Conclusion:**

Clinicians should consider the possibility of TTP when a patient presents with acute stroke-like symptoms and thrombocytopenia, especially in an emergency room, either with or without new stroke lesions on the brain CT and MRI.

## Background

Thrombotic thrombocytopenic purpura (TTP) is a rare and fatal thrombotic microangiopathy-based hematologic disease characterized by severe thrombocytopenia, microangiopathic hemolytic anemia, and end organ ischemia (particularly brain, heart and kidney) associated with microvascular platelet-rich thrombi formation [1]. Stroke with lesions on brain computed tomographic (CT) / magnetic resonance imaging (MRI), or transient ischemic attack (TIA), has been reported as atypical neurological manifestations of TTP in some cases [2–5]. However, TTP presenting as acute stroke mimics without new stroke lesions on the brain MRI has not been reported yet.

## Case presentation

A 65-year-old male patient encountered a sudden onset of slurred speech at 8:30 in the morning (Day 0), which was breifly resolved at noon, then relapsed at 20:00 on the same day and persisted, accompanied with right-sided weakness, facial paralysis and confusion (National Institute of Health Stroke Score (NIHSS) = 11). He presented to a local hospital and completed a brain CT scan with a normal result. He was detected with an unexplained thrombocytopenia with a platelet count of 19 × 10^9^/L on Day 0 at the local hospital, and was not given intravenous thrombolysis or any antiplatelet therapy. The patient was then transferred to our hospital the next morning (Day 1) and admitted to the department of neurology. He reported a history of anemia 4 years ago, which was rectified without further examination. He had no previous personal or familial history of stroke or thrombosis.

The assessment at the time of admission showed disorientation, dysarthria, right-sided facial paralysis and hemiplegia, and hypermyotonia of the left-sided extremities. The patient also had several bruises on the left leg and both hands.

**Fig. 1:**
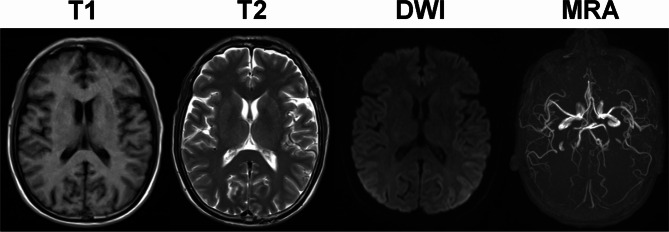
Brain MRI (T1, T2, DWI, MRA) scan on Day 2 did not show new infarct

**Fig. 2:**
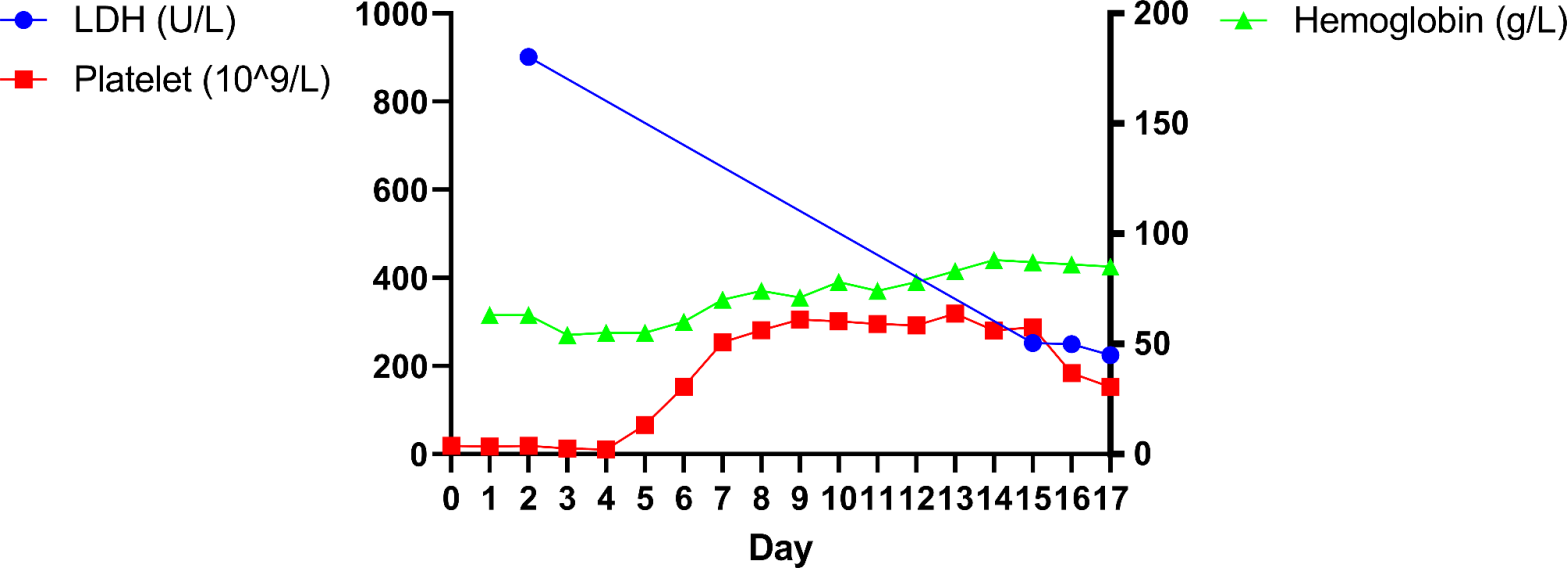
LDH, platelet count, and hemoglobin level versus time

The brain MRI and diffusion-weighted imaging (DWI) on Day 2 were both negative without presenting new ischemic lesions (Fig. 1). There was also no evidence of thrombosis on the magnetic resonance angiography (MRA) (Fig. 1). Laboratory tests on Day 1 showed a platelet count of 17 × 10^9^/L, a hemoglobin level of 63 g/L, a serum total bilirubin level of 25.7µmol/L, and a direct bilirubin level of 8.3µmol/L. On Day 2, a low ADAMTS-13 activity (below 1%) and a high lactate dehydrogenase (LDH) level (901 U/L) were detected, and the peripheral blood smear was found to be > 5% schistocytes. Based on the results above, the patient was diagnosed with TTP. Then, plasma exchanges (PE) with fresh frozen plasma were conducted once a day from Day 3 to Day 7, and then every other day for twice. The platelet count was normalized to 153 × 10^9^/L on Day 6. The hemoglobin level was increased to 88 g/L on Day 14. Both indicators were gradually improved (Fig. 2). The percentage of schistocytes in the peripheral blood smear decreased to 2.2% (11/500) on Day 14. The LDH level decreased to 251.7 U/L, while the serum total bilirubin level and direct bilirubin level decreased to normal on Day 15. The renal function remained normal consistently. In addition, bilateral cephalic vein thrombosis and plexus venosus leg muscle thrombosis were detected on Day 13 by ultrasonography, while no deep vein thrombosis was found on Day 2. Therefore, rivaroxaban was added to the treatment therapy on Day 13. The facial paralysis, hemiplegia, and dysarthria symptoms were rapidly resolved undergoing plasma exchanges therapy, followed by recovery of cognitive impairment. The symptoms and positive signs of the nervous system above were almost completely resolved 14 days after initiation of the PE therapy (NIHSS = 0).

## Discussion and conclusions

In this report, we described a rare case of atypical TTP presented as stroke mimics. This is the first report highlighting TTP with rapid-onset and lasting stroke symptoms but without positive findings on the brain MRI. The patient presented with typical acute ischemic stroke symptoms including sudden-onset dysarthria, right-sided facial paralysis and hemiplegia. However, his CT and MRI scans (including DWI and MRA) were negative without showing any new ischemic lesions. The laboratory findings including severe thrombocytopenia, elevated LDH level, and severe deficiency in ADAMTS13 were typical for TTP. The patient’s stroke-like symptoms were relieved soon after administrating the PE therapy, which also supported the diagnosis of TTP.

TTP is specifically related to a severe deficiency in ADAMTS13 (a disintegrin and metalloprotease with a thrombospondin type 1 motif, member 13) (activity < 10%). ADAMTS13 is a metalloprotease that inhibits the von Willebrand factor–dependent platelet aggregation and is the only biologic marker specific for TTP [1]. Commonly reported neurological complications of TTP encompass altered consciousness, confusion, seizures, headaches, stroke, and TIA [6]. Given that the patient did not exhibit any typical epilepsy symptoms during the course of the disease, we opted not to conduct an electroencephalogram. However, seizures should be considered as a common neurological manifestation of TTP [7]. The main therapy of TTP is therapeutic PE [1].

Stroke mimics are a group of non-vascular disorders that mimic the clinical signs of real acute ischemic stroke, commonly due to metabolic/toxic disturbances (such as hypoglycemia, hyponatraemia, hypokalaemia, and alcohol), hemiplegic migraine, seizures, syncope, sepsis, psychogenic, peripheral vestibular disease and functional disorder [8]. It was reported that approximately 12.7–26.8% of the patients referring to the emergency department or hospitalized for stroke-like symptoms were stroke mimics [9–11]. Though rapid treatment is essential for acute stroke since many stroke therapies are time-dependent (such as thrombolysis or surgical thrombectomy), efforts should still be made to avoid inappropriate therapeutic attempts in patients with diagnosis other than stroke. Primary mistaken diagnosis may result in delayed or inappropriate treatment that poses an adverse effect on the prognosis. Patients with stroke mimics are of complex etiology and in need of individualized treatments in order to address their actual causes. In our case, the stroke-mimic symptoms may be linked to thrombotic microangiopathy in the brain caused by TTP, potentially leading to focal ischemia and hypoxia. However, further investigations are required to elucidate the precise mechanism. Rapid recognition of TTP is crucial for initiating appropriate treatment, especially PE, otherwise it can be life-threatening.

In sum, TTP can present as acute stroke mimics without evidence of stroke lesions on the brain MRI, which is rare and has not been reported before. Though challenging for neurologists, a timely and proper diagnosis is crucial to initiating the right therapy and saving life. TTP should be taken into consideration when a patient with acute stroke-like symptoms and severe thrombocytopenia is presented in a clinical setting, especially in an emergency room, either with or without new stroke lesions on the brain CT and MRI.

## Data Availability

All data generated or analysed during this study are included in this published article.

## Abbreviations

TTP: Thrombotic thrombocytopenic purpura
CT: Computed tomography
MRI: Magnetic resonance imaging
TIA: Transient ischemic attack
NIHSS: National Institute of Health Stroke Score
DWI: Diffusion-weighted imaging
MRA: Magnetic resonance angiography
ADAMTS13: A disintegrin and metalloprotease with a thrombospondin type 1 motif, member 13
